# Effect of Emotional Intelligence and Psychosocial Risks on Burnout, Job Satisfaction, and Nurses’ Health during the COVID-19 Pandemic

**DOI:** 10.3390/ijerph17217998

**Published:** 2020-10-30

**Authors:** Ana Soto-Rubio, María del Carmen Giménez-Espert, Vicente Prado-Gascó

**Affiliations:** 1 Personality, Assessment and Psychological Treatments Department, Faculty of Psychology, University of Valencia, 46010 Valencia, Spain; ana.soto@uv.es; 2 Department of Nursing, Faculty of Nursing and Chiropody, University of Valencia, 46010 Valencia, Spain; 3 Social Psychology Department, Faculty of Psychology, University of Valencia, 46010 Valencia, Spain; vicente.prado@uv.es

**Keywords:** burnout, COVID-19, emotional intelligence, health, job satisfaction, nurses, psychosocial risks

## Abstract

Nurses are exposed to psychosocial risks that can affect both psychological and physical health through stress. Prolonged stress at work can lead to burnout syndrome. An essential protective factor against psychosocial risks is emotional intelligence, which has been related to physical and psychological health, job satisfaction, increased job commitment, and burnout reduction. The present study aimed to analyze the effect of psychosocial risks and emotional intelligence on nurses’ health, well-being, burnout level, and job satisfaction during the rise and main peak of the COVID-19 pandemic in Spain. It is a cross-sectional study conducted on a convenience sample of 125 Spanish nurses. Multiple hierarchical linear regression models were calculated considering emotional intelligence levels, psychosocial demand factors (interpersonal conflict, lack of organizational justice, role conflict, and workload), social support and emotional work on burnout, job satisfaction, and nurses’ health. Finally, the moderating effect of emotional intelligence levels, psychosocial factors, social support, and emotional work on burnout, job satisfaction, and nurses’ health was calculated. Overall, this research data points to a protective effect of emotional intelligence against the adverse effects of psychosocial risks such as burnout, psychosomatic complaints, and a favorable effect on job satisfaction.

## 1. Introduction

Nurses play a crucial role in health systems, constituting approximately 60% of the professionals in this field [1,2]. Nurses’ contribution to global health is undisputed and investing in improving their quality of life benefits society [3,4]. Investing in the working conditions and quality of life of nurses benefits not only their well-being but also their performance and, by extension, the entire health care system [5].

On a daily basis, health professionals are exposed to situational factors that can make their work difficult, which include the so-called psychosocial factors. Psychosocial risks have been related to health problems [6], work accidents [7,8,9], low job satisfaction [10], low work engagement [11], burnout [12,13], and work-related stress [14]. The latter is related to an increase in physiological pain and cardiovascular problems, a reduction in social interaction and the ability to concentrate at work, and a higher incidence of mental illness such as depression and anxiety [15]. At the same time, the proper management of psychosocial risks helps to increase productivity [10,16,17], prevent accidents and absenteeism [8,10], and promote employees’ well-being [18].

Physicians and nurses constitute a professional group that meets high work demands, great responsibility, and strong commitment [19]. According to Kasarek [20], psychological demands have a nearly quantitative conception: volume of work in relation to the time available to do it (time pressure) and the interruptions that force to leave the tasks momentarily and to return to them later. In addition to these two dimensions (time pressure and number of interruptions), the third dimension of this model is social support [21]. Workers exposed to high demands, little control, and scarce social support present twice the risk of morbidity and mortality from cardiovascular disease than those with low demands, much control, and strong social support [21]. In addition to social support, the following stand out as frequent psychosocial risks for this group: (1) Workload, which may refer to qualitative or quantitative. Quantitative workload refers to the number of activities to be performed in a given period. In contrast, qualitative workload refers to the difficulty of the task and the volume of information to be processed in relation to the time available [22]. A high workload has been associated with low levels of well-being and higher risks of health problems [23]. (2) Lack of organizational justice, which is the extent to which employees perceive they are treated unfairly in their workplace and the perception of the absence of reciprocity in social exchanges [24,25]. Low organizational justice is a potential risk factor for poor physical and psychological health among employees [24,26]. (3) Emotional work refers to the effort, planning, and control necessary to express the organizationally desirable emotions during interpersonal transactions [27]. It includes dealing with intense feelings at work [28]. Previous research has shown that problematic distress levels were 38% more likely for workers reporting high emotional work [28]. (4) Role conflict happens when an employee is being given work tasks without enough resources to complete them and receive contradictory requests from different people. Previous research has shown that problematic distress levels were 53% more likely for workers reporting role conflict [28]. (5) Finally, interpersonal conflicts happen when workers perceive conflicts are coming from the hospital management, colleagues, patients, or relatives of the patient. Interpersonal conflicts have been associated with health problems, particularly to depression [29].

Prolonged stress at work can lead to the burnout syndrome [13,30]. The burnout syndrome has been defined by Maslach, Schaufeli, and Leiter [13] as a prolonged response to chronic emotional and interpersonal stressors at work. It is defined by the three dimensions of burnout, cynicism, and inefficiency [13]. It is associated with different consequences such as absenteeism [30], psychosomatic problems, lower employee performance [31], and greater depression and drug consumption [32]. It is characterized by affecting service sector employees such as teachers, police officers, nurses, and doctors [31] with a prevalence in this sector between 35% and 40% [33,34].

One of the most relevant and influential theoretical models is the demand-control model of Robert Karasek [20]. It explains labor stress in terms of the balance between the job’s psychological demands and the level of control of the worker over these. From this model, the worker’s health or well-being will depend on the balance between the demands of the job and the worker’s available resources.

The effects of psychosocial risks can affect psychological and physical health through psychophysiological mechanisms activated by stress [35]. The excess of demands and emotional requirements, along with limited personal or material resources to cope with them, can produce this negative psychological state in workers [36].

One of the professional sectors with the greatest tendency to suffer pathologies related to psychosocial factors is health professionals [36]. The effects on health professionals of psychosocial risk factors are usually manifested as dissatisfaction with their work, greater perception of health problems, and higher levels of emotional stress or social problems related to their profession [37]. All of this, in turn, leads to a decline in the quality of care for patients and their families. This reality is even more negative in the case of nurses [38] due, in large part, to the high emotional burden of continued contact with patients’ suffering and pain.

It is necessary to emphasize that the good handling of the psychosocial risks appears to be associated with elements of satisfaction in the work that act as a factor of protection in front of the risks that suppose the labor stress in general. Job satisfaction can be defined as the extent to which people like their jobs and find them satisfying [39] or how the worker perceives that their needs are met by their job [40]. Several theoretical models explain the factors that lead to high job satisfaction.

Another important protective factor against psychosocial risks is interpersonal skills and understanding each other (emotional intelligence) [41]. Mayer and Salovey [42] define emotional intelligence as the ability to recognize, understand, and regulate one’s own and other people’s emotions, discriminate between them, and use this information to guide thoughts and actions.

In order to establish an adequate therapeutic relationship with patients and, at the same time, to implement effective self-care strategies, the emotional management of the nurse is crucial, both in relation to their ability to understand their own emotions and those of others, and express them appropriately, as well as their ability to put themselves in the patient’s shoes [43,44,45].

Emotional intelligence is a prerequisite for key skills such as communication and empathy, sensitivity, creativity, self-awareness, self-control, and assertiveness [46]. Studies such as Rego et al. [47] suggest that emotional intelligence is one of the most important skills that lead nurses to be more respectful of their patients and provide them with more information about treatments and their consequences. The Simpson and Keegan [48] study has shown that emotional intelligence allows for open communication between the nurse, patient, and family, allowing them to share feelings and emotions. With greater emotional intelligence, the nurse is more willing to listen to patients, help them, and care for them [48].

The scientific literature considers the importance of emotional intelligence in nurses [49], reflecting strong implications of emotional intelligence for nurses themselves, their physical and psychological health, job satisfaction and the quality of the nurse-patient relationship, and increased teamwork and better interprofessional relationships [50]. This ability to manage one’s own emotions and interpret others’ emotions helps nurses combat stress, which contributes positively to both their own health and the patient’s health [51]. Some studies, such as Codier, Muneno, Franey, and Matsuurza [52], measured emotional intelligence in clinical practice and found positive relationships between emotional intelligence and performance, increased commitment to the organization, and job satisfaction of professionals. Some studies relate emotional intelligence to reduced burnout, improved performance, and job loyalty [53].

Working conditions, and the consequences that arise from them, can be greatly affected by the economic and social context [54], especially when events that affect the entire population arise, such as economic crises or, in this case, health emergencies or pandemics, such as that caused by COVID-19. The World Health Organisation (WHO) recognized it as a global pandemic on 11 March 2020 [55]. As of 23 September 2020, 31,658,573 cases of the disease were reported worldwide, including 971,869 deaths [56]. Health care providers are particularly vulnerable to emotional distress in the current pandemic, given their risk of exposure to the virus, concern about infecting and caring for their loved ones, shortages of personal protective equipment (PPE), longer work hours, and involvement in emotionally and ethically fraught resource-allocation decisions [57]. In Spain, as of 23 September 2020, there have been 682,267 confirmed cases, of which 30,904 have died [56]. The alarming health situation generated by the COVID-19 pandemic has meant enormous overexertion of all health personnel at the national level, including nurses, who have had to face physical, psychological, emotional, and social demands in a situation where resources are not always available, and the uncertainty of the evolution of the pandemic has been present, with the main peak of the pandemic occurring between late March and early April 2020 [58].

Despite the importance of psychosocial risks and intelligence in the development of burnout, job satisfaction, and worker health and well-being, few studies address this issue specifically in nurses, and even more so at a time of such need as a pandemic. After conducting a review of the literature, we were unable to observe any studies focused on nurses that analyzed the psychosocial risks of this group and their relation to emotional intelligence, considering burnout, job satisfaction, health, and well-being during a global health crisis.

Therefore, the present study aims to fill this gap in the literature by offering an analysis of the effect that psychosocial risks and emotional intelligence can have on the health, well-being, burnout level, and job satisfaction in Spanish nurses during the rise and main peak of the COVID-19 pandemic in Spain, from late March to early April 2020.

The work of the nurses is always fundamental. In a pandemic situation, their work is even more necessary and critical, while the risks and situations to which they are usually exposed are heightened. We have now been in a pandemic situation for seven months, and it does not seem to be coming to an end.

Given the importance of relationships, emotional intelligence, and psychosocial risks in preventing burnout, increasing job satisfaction, and improving health, this study aims to analyze these relationships in the Spanish context during the pandemic. This research is meant to better understand this reality and contribute as much as possible to the care of this important health sector to which we so much need and owe in the current circumstances.

## 2. Materials and Methods 

### 2.1. Sample 

This study was carried out in a convenience sample of 125 nurses from three public hospitals in Valencia, Spain. The sample of nurses from the three selected hospitals was representative of the Spanish nursing sample, considering a population of 316,094 with a 95% confidence interval and an alpha error of 9% in a sample of 119 nurses. This study respected the Declaration of Helsinki (World Medical Association, 2013) with particular emphasis on the anonymization of the data collected, confidentiality, and non-discrimination of participants. This study was authorized by the Research Ethical Committees of the hospitals included in the study (Ethical Committee of Research with Medicine Ceim 128/19, Ethical Committee of Research with Medicine 2020/00096/PI, Approval of the investigation commission in care of the Valencia Health Department—Clinico Malvarrosa). The data was collected from March to April at the peak of the COVID-19 pandemic in Spain. The participants completed the self-completed questionnaires online, which took around 35 min. The inclusion criteria were: (1) to be a nurse working in one of the participating hospitals, and (2) to provide their informed consent to participate. Finally, 156 questionnaires were distributed, and 125 were completed by obtaining a response rate of 80%.

### 2.2. Method and Variables

This study is a cross-sectional one. The variables included were emotional intelligence, psychosocial risks (social support, workload, lack of organizational justice, emotional work, role conflict, and interpersonal conflicts), burnout, job satisfaction, and psychosomatic health problems. These variables were measured using the following instruments.

- The Trait Meta-Mood Scale (TMMS-24) was used to assess emotional intelligence [59]. It is a reduced version of the TMMS-48 [60] using 24 items grouped in three dimensions: attention, clarity, and repair. The answers are recorded on a five-point Likert scale with anchors of 1 (I do not agree at all) and 5 (I completely agree). Examples of the items are: “I pay much attention to my feelings” (attention), “I am usually very clear about my feelings” (clarity), and “Although sometimes I am sad, I have mostly an optimistic outlook” (repair). This instrument presented adequate psychometric properties in previous studies (all Cronbach’ s alphas > 0.85) [60,61] as well as in this study (all Cronbach’ s alphas > 0.88).
- The UNIPSICO Battery (*Unidad de Investigación Psicosocial de La Conducta Organizacional*) [22,62] was used to assess psychosocial risks. This battery assesses the psychosocial risks of workers considering three factors: demands, resources, and consequences. All items are answered using a five-point Likert scale ranging from 0 (never) to 4 (very often: every day). This instrument has presented adequate psychometric properties in previous studies (Cronbach’ s alphas > 0.86) [22] and the present one. More specifically, the scales of the UNIPSICO Battery used in this study were:
  1. Interpersonal conflicts scale. It assesses the frequency with which workers perceive conflicts related to management, supervisors, colleagues, other employees of the hospital, patients, and patient’s families. It is composed of six items (e.g., “How often do you have conflicts with your peers?”). The Cronbach alpha for the sample of study is Cronbach’s α = 0.43.
  2. Lack of organizational justice scale. It assesses the perceived lack of reciprocity in social exchanges in the work environment. It is composed of five items (e.g., “I work very hardly compared to what I receive in return”). The Cronbach alpha for the sample of study is Cronbach’s α = 0.88.
  3. Role conflict scale. It assesses the situations in which a person cannot simultaneously satisfy the conflicting role expectations in which he or she is involved. It is used to assess psychosocial demands. It is comprised of five items (e.g., “I receive incompatible demands from two or more people”). The Cronbach alpha for the sample of study is Cronbach´s α = 0.78.
  4. Workload scale. This scale assesses quantitative and qualitative workload. The quantitative workload refers to the number of activities to be carried out in a given time. In contrast, the qualitative refers to the difficulty of the task and the volume of information to be processed in relation to the time available. It is used to assess psychosocial demands, and it is composed of six items (e.g., “Do you have insufficient time to complete your work, do you think you have to do a job that is too difficult for you?”). The Cronbach alpha for the sample of study is Cronbach´s α = 0.77.
  5. Social support scale. It assesses the worker’s perception of social support received by hospital management, direct supervisors, and peers. It assesses the perception of emotional and technical support. It comprises of six items (e.g., “I feel appreciated at work by the center’s managers?”). The Cronbach alpha for the sample of study is Cronbach´s α = 0.81.
  6. Job satisfaction scale. It measures the positive or pleasant emotional state resulting from the subjective perception of the person’s work experiences. The scale comprises six items (e.g., “Are you satisfied with the salary or pay you receive?”). The Cronbach alpha for the sample of study is Cronbach´s α = 0.78.
  7. Psychosomatic health problems scale. It assesses the frequency of psychosomatic problems related to anxiety arising from perceived sources of stress at work. It includes nine items (e.g., “Have you had pain or discomfort in your stomach?” “Have you had difficulty sleeping?”). Besides these nine items, the scale presents two extra ones asking the worker how often they have needed specialist support in the last year to overcome a personal crisis related to their work and how often they use medication to treat psychosomatic health problems related to work. The Cronbach alpha for the sample of study is Cronbach´s α = 0.88.
- The Frankfurt Emotional Work Scale (FEWS) was used to assess emotional work. More specifically, an adapted version of this questionnaire [27], included in the UNIPSICO (4–5) battery, was applied. This scale assesses the effort that the worker has to invest in exhibiting the appropriate emotions at work and inhibiting the inappropriate ones [27]. The adaptation includes eleven items (e.g., “How often do you need to repress your emotions to appear neutral/quiet at work?”). The Cronbach alpha for the sample of study is Cronbach´s α = 0.56.
- The Questionnaire for the Assessment of Workplace Burnout Syndrome (CESQT) [63] was used to assess burnout syndrome. Burnout syndrome is characterized by the loss of illusion at work, emotional and physical exhaustion, and negative attitudes toward others or the organization (indifference, or even anger) [64]. It comprises fifteen items, scored from 0 (never) to 4 (very often: every day) points Likert scale (e.g.,” I see my work as a source of personal satisfaction”). The Cronbach alpha for the sample of study is Cronbach´s α = 0.89.

### 2.3. Statistical Analyses

Multiple hierarchical linear regression models were calculated considering emotional intelligence levels, psychosocial demand factors (interpersonal conflict, lack of organizational justice, role conflict, and workload), social support and emotional work on burnout, job satisfaction, and nurses’ health during the COVID-19 pandemic. In the case of hierarchical regression models, two steps were considered for the prediction of burnout, job satisfaction, and nurses’ health: interpersonal conflict, lack of organizational justice, role conflict and workload, social support (step 1), and the three dimensions of the TMMS24 questionnaire (step 2).

Finally, the moderating effect of the independent variables (TMMS24 dimensions, interpersonal conflict, lack of organizational justice, role conflict, workload, social support, and emotional work) on the criterion variables psychosocial risks (job satisfaction, health problems, and burnout syndrome) was assessed. For this purpose, the possible interactions between the variables were analyzed according to the theoretical framework. The moderation effect is demonstrated when the corrected confidence interval (95%) of the indirect effect does not include zero [65]. All these analyses were performed using the Statistical Package for the Social Sciences (SPSS) version 24.0. 

## 3. Results

### 3.1. Sociodemographic Characteristics of the Sample

The participants’ age range was 24 to 63 (M = 43.37, SD = 11.58), and 79.1% of them were women. Depending on the employment situation, 43% (54) are temporary workers, while 57% (71) are permanent workers. The educational levels of the nurses in the sample were: degree 69% (86), master 26% (33), and doctorate 5% (6).

### 3.2. Hierarchical Regression Models Based on Emotional Intelligence and Psychosocial Risks on Burnout, Job Satisfaction, and Nurses’ Health

The predictive value of emotional intelligence and psychosocial risks was analyzed using hierarchical regression models. The criterion variables were burnout, job satisfaction, and psychosomatic health problems. In this line, two differentiated steps were established in the model. First, the variable psychosocial risks were introduced in the first step. Later, the variable emotional intelligence was included in the second step.

#### 3.2.1. Psychosocial Risks and Emotional Intelligence Dimensions Predict Burnout

In the prediction of the burnout (R^2^_adjusted_ = 0.50, *p* ≤ 0.001), two steps were established in the model. First, psychosocial risks were entered (social support, workload, lack of organizational justice, emotional work, interpersonal conflict, and role conflict) (step 1) (ΔR^2^ = 0.46, *p* ≤ 0.001). Afterward, the emotional intelligence components were included (step 2) (ΔR^2^ = 0.08, *p* ≤ 0.001). Regarding psychosocial risks, the significant positive predictors of burnout were emotional work (Β = 0.16, *p* ≤ 0.05), interpersonal conflict (Β = 0.17, *p* ≤ 0.05), and role conflict (Β = 0.44, *p* ≤ 0.001). With respect of the emotional intelligence components, the significant negative predictor of burnout was emotional repair (Β = −0.26, *p* ≤ 0.001) (Table 1).

#### 3.2.2. Psychosocial Risks and Emotional Intelligence Dimensions Predict Job Satisfaction

Regarding the prediction of job satisfaction (R^2^_adjusted_ = 0.41, *p* ≤ 0.001), two steps were established in the model. First, the psychosocial risks were entered (social support, workload, lack of organizational justice, emotional work, interpersonal conflict, and role conflict) (step 1) (ΔR^2^ = 0.42, *p* ≤ 0.001). Then, the emotional intelligence components were included (step 2) (ΔR^2^ = 0.03, *p* = 0.08). From the psychosocial risks, social support (Β = 0.44, *p* ≤ 0.001) is a significant positive predictor of job satisfaction. Workload (Β = −0.23, *p* ≤ 0.01) is a significant negative predictor of job satisfaction. Finally, from the emotional intelligence components, emotional repair is a significant positive predictor of job satisfaction (Β = 0.20, *p* ≤ 0.05) (Table 1).

#### 3.2.3. Psychosocial Risks and Emotional Intelligence Dimensions Predict Nurses’ Psychosomatic Health Problems

In the prediction of the nurses’ psychosomatic health problems (R^2^_adjusted_ = 0.32, *p* ≤ 0.001), two steps were established in the model. First, the psychosocial risks were entered (social support, workload, lack of organizational justice, emotional work, interpersonal conflict, and role conflict) (step 1) (ΔR^2^ = 0.34, *p* ≤ 0.001). Afterward, the emotional intelligence components were included (step 2) (ΔR^2^ = 0.04, *p* = 0.08). Regarding the psychosocial risks, workload (Β = 0.27, *p* ≤ 0.01), and role conflict (Β = 0.29, *p* ≤ 0.01) are significant positive predictors of the nurses’ psychosomatic health problems. Regarding the emotional intelligence components, emotional attention (Β = 0.22, *p* ≤ 0.05) is a significant positive predictor of the nurses’ psychosomatic health problems (Table 1).

In general, it can be observed that psychosocial risks and emotional intelligence predict 50% of the variance in burnout, 41% of the variance observed in job satisfaction, and 32% of the variance observed in nurses’ psychosomatic health problems.

### 3.3. Moderation of Emotional Intelligence on Psychosocial Risks

The moderation analysis between variables based on the scientific literature’s recommendations is significant [65] when the corrected confidence interval (95%) of the indirect effect does not include zero.

#### 3.3.1. Moderation of Emotional Intelligence on the Effect of Psychosocial Risks on Burnout

**Figure 1:**
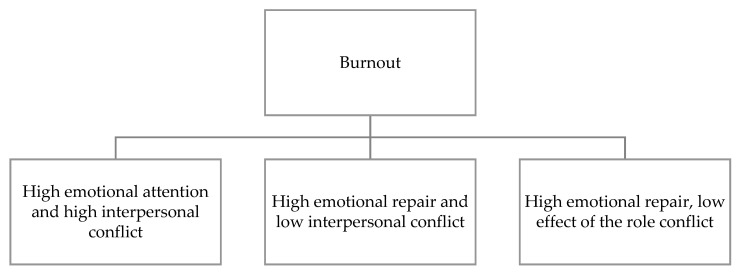
Moderation of emotional intelligence on the effect of psychosocial risks on burnout.

A moderating effect of emotional intelligence was observed on the effect of interpersonal conflict on burnout (Figure 1). Specifically, this moderation was observed in the emotional attention and emotional repair components. High emotional attention increased the predictive power of interpersonal conflict on burnout. At the same time, high emotional repair reduced the predictive power of interpersonal conflict on burnout.

Finally, the emotional repair component also moderated the effect of role conflict on burnout. High emotional repair reduced the predictive power of role conflict on burnout (Table 2).

#### 3.3.2. Moderation of Emotional Intelligence on the Effect of Psychosocial Risks on Job Satisfaction

**Figure 2:**
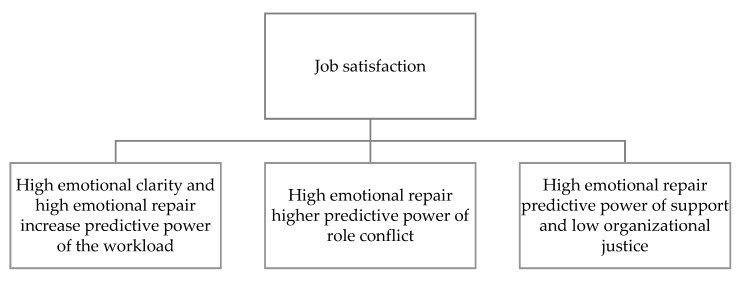
Moderation of emotional intelligence on the effect of psychosocial risks on job satisfaction.

A moderating effect of emotional intelligence has also been reported in the effect of workload on nurses’ job satisfaction (Figure 2). This effect is observed in emotional repair and emotional clarity components, where high levels of these two variables increase the predictive power of workload on job satisfaction. The emotional repair component of emotional intelligence also moderates the effect of role conflict, where high emotional repair increases the predictive power of role conflict on job satisfaction. On the other hand, the emotional repair component of emotional intelligence moderates the predictive power of social support and lack of organizational justice on job satisfaction, where high emotional repair increases the effect of social support on job satisfaction while reducing the effect of lack of organizational justice on job satisfaction (Table 2).

#### 3.3.3. Moderation of Emotional Intelligence on the Effect of Psychosocial Risks on Psychosomatic Health Problems

**Figure 3:**
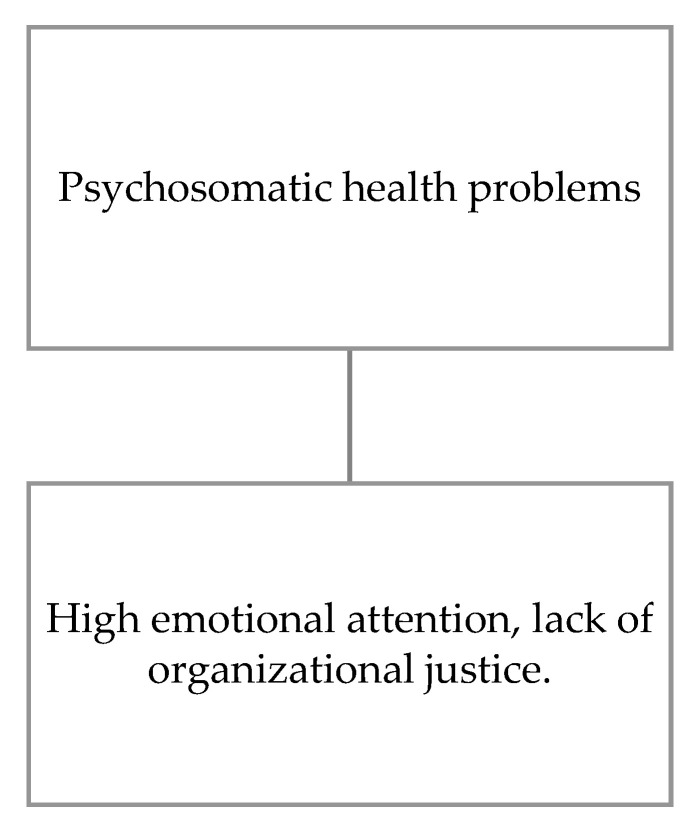
Moderation of emotional intelligence on the effect of psychosocial risks on psychosomatic health problems.

Concerning the moderation of emotional intelligence on the effect that psychosocial risks have on psychosomatic nurses’ health problems (Figure 3), a moderating effect of emotional attention is observed in the predictive power of a lack of organizational justice, where high emotional attention increases the effect of inferior organizational justice on psychosomatic problems (Table 2).

## 4. Discussion

In general, the scientific literature shows the importance of psychosocial risks and emotional intelligence in the health and well-being of workers [6], their satisfaction with their work [10], and even their risk of developing burnout syndrome [12,13]. Considering psychosocial risks are relevant for workers in general, particularly for service sector workers, as they are exposed to high emotional demands and very high levels of stress and responsibility [19], as is the case of teachers and health professionals. This paper has focused on a very particular sector of health professionals who play a central role in the health system. Furthermore, it has focused on studying the relationships between these variables in this group at a time of particular need and vulnerability: the global health crisis caused by the COVID-19 pandemic [55]. This study’s data has been collected from nurses active in the Spanish health system at the time of the pandemic’s most significant peak in Spain, that is, in late March and early April 2020. These research circumstances offer a unique and privileged opportunity to understand better how these variables relate to nurses during a pandemic in order to be able to design future research programs aimed at improving health and well-being in this sector at the time when they need it most. The crisis generated by the COVID-19 is unfortunately not yet under control, neither in Spain nor in the world, and it is necessary to understand how different variables that influence the well-being of health professionals are related in this context of a pandemic.

Based on the results of this study, and in line with previous scientific literature [37,41,44,52], it can be said that psychosocial risks and emotional intelligence largely predict burnout, job satisfaction, and the health of nurses in which the latter is understood as psychosomatic health problems. Simultaneously, a moderation of emotional intelligence has been observed in the effect of psychosocial risks on burnout, job satisfaction, and nurses’ health. This moderation makes sense if considered in relation to previous research, highlighting the importance of nurses’ abilities to perceive and regulate their emotions while being aware of and empathizing with others’ emotions. The emotional skills of health professionals not only favor a good therapeutic relationship with the patient and greater satisfaction with their work but also act as a protective factor against the adverse effects of psychosocial risks such as burnout and occupational stress, which, in turn, has been closely related to health problems, including psychosomatic complaints [41,47,48,50,51,52,53].

In general, the data from this research point to a protective effect of emotional intelligence against the adverse effects of psychosocial risks such as burnout and psychosomatic complaints and a favorable effect on job satisfaction. However, in this particular case of emotional attention as a component of emotional intelligence, the moderating effect may be harmful [61]. It has been observed that emotional attention increases the effect of interpersonal conflicts on burnout [66]. This result could make sense if we think that paying too much attention to emotional aspects in a work environment where there are interpersonal conflicts can accentuate the negative effect that these conflicts have on the worker such as the nurses. This contributes to a greater vulnerability to burnout [67,68]. Something similar is observed in the effect of a lack of organizational justice on job satisfaction: the effect of one on the other is accentuated by emotional attention. Again, our data seem to indicate that a high level of emotional attention could be making nurses more vulnerable, in this case, negatively impacting their job satisfaction.

These data are particularly important if we think that those skills that favor the establishment of a good therapeutic relationship with the patient, such as emotional attention, may, in turn, be a risk factor for some psychosocial risks, such as interpersonal conflicts or a lack of organizational justice. However, it should be noted that this is observed specifically with the emotional attention component of emotional intelligence. However, the emotional repair component stands out as an emotional intelligence element to be enhanced to prevent the possible adverse effects of psychosocial risks on nurses, specifically those related to burnout, psychosomatic complaints, and job satisfaction [69]. We believe that these data can help ensure nurses’ health and psychological well-being, especially in situations of such complexity and crucial importance as a health crisis of the magnitude of a pandemic, such as the one we are currently experiencing due to COVID-19.

This study has some limitations, however. On the one hand, it is a transversal study, making it difficult to establish causal relationships between the variables. Future research of a longitudinal nature could shed more light on this question as to how the relationships between the variables evolve. At the same time, it is carried out in a particular context, the Spanish language. Future research could try, on the one hand, to study the existing relationships between the variables of the present study in a context other than Spanish, so that cultural and contextual differences can be taken into account. Finally, the results from this study have been obtained in the middle of a pandemic, so their generalizability to other contexts must be done with caution. Nevertheless, the main findings go in line with those found in previous studies in other populations [37,41,44,52]. Despite its limitations, this research data provide a glimpse into a critical issue, such as the effect of psychosocial risks on nurses’ health and well-being. Furthermore, the data reflects these relationships at a time of extraordinary crisis in Spain, as they have been collected at the highest peak of the COVID-19 pandemic within the country. We believe that this study’s data are an excellent opportunity to study this critical issue for nurses, the health system, and society in a context as particular and difficult to study as the peak of a pandemic. We hope that the data provided here can help ensure nurses’ well-being in these and future circumstances.

## 5. Conclusions

The emotional intelligence of nurses, in particular the emotional attention dimension, can be a risk factor for some psychosocial risks, such as interpersonal conflicts or lack of organizational justice. However, the emotional repair component stands out as an element of emotional intelligence that should be enhanced to prevent the possible adverse effects of psychosocial risks on nurses, specifically those related to burnout, psychosomatic complaints, and job satisfaction. We believe that these data can contribute to the development of intervention programs that help ensure nurses’ health and psychological well-being, especially in situations of this complexity and crucial importance as a health crisis of the magnitude of a pandemic we are currently experiencing due to COVID-19.

## Figures and Tables

**Table 1:** Hierarchical regression models based on emotional intelligence and psychosocial risks on burnout, job satisfaction, and nurses’ health.

Variable	Burnout		Job Satisfaction		Nurses’ Health	
Predictors	ΔR^2^	Β	ΔR^2^	Β	ΔR^2^	Β
Step 1	0.46 ***		0.42 ***		0.34 ***	
Social Support		−0.13		0.45 ***		−0.14
Workload,		−0.05		0.23 **		0.26
Lack of organizational justice Emotional work Interpersonal conflict Role conflict		0.11 0.09 0.24 *** 0.45 ***		−0.04 0.05 0.04 −0.13		0.04 ** 0.05 0.34 *** −0.12
Step 2	0.08 ***		0.03		0.04	
Social Support		−0.11		0.44 ***		−0.17
Workload,		−0.06		−0.23 **		0.27 **
Lack of organizational justice Emotional work		0.11		−0.04		0.02
Emotional work		0.16 *		0.01		0.08
Interpersonal conflict		0.17 *		0.08		−0.11
Role conflict		0.44 ***		−0.12		0.29 **
Emotional Attention		−0.03		−0.03		0.22 *
Emotional Clarity		−0.05		0.00		−0.18
Emotional Repair		−0.26 ***		0.20 *		0.07
Total R^2^_adjusted_	0.50 ***		0.41 ***		0.32 ***	

Note. * *p* ≤ 0.05, ** *p* ≤ 0.01, *** *p* ≤ 0.001.

**Table 2:** Moderation of emotional intelligence on the effect of psychosocial risks on burnout, job satisfaction, and psychosomatic health problems.

Moderator Effect on Nurses’ Burnout							
	Coefficients	SE	t	*p*	R^2^	F (*p*)	Confidence Interval
Emotional Attention > Interpersonal Conflict	0.33	0.16	2.09	0.04	0.22	6.79 (0.00)	(0.02)–(0.64)
Emotional Repair > Interpersonal Conflict	−0.42	0.16	−2.63	0.01	0.28	15.54 (0.00)	(−0.73)–(−0.10)
Emotional Repair > Role Conflict	−0.33	0.13	−2.59	0.01	0.50	39.96 (0.00)	(−0.58)–(−0.08)
**Moderator Effect on Nurses’ Job Satisfaction**							
	Coefficients	SE	t	*p*	R^2^	F (*p*)	Confidence Interval
Emotional Clarity > Workload	−0.40	0.15	−2.65	0.01	0.23	7.26 (0.00)	(−0.71)–(−0.10)
Emotional Repair > Workload	−0.30	0.13	−2.40	0.02	0.26	14.50 (0.00)	(−0.55)–(−0.05)
Emotional Repair > Role Conflict	−0.57	0.20	−2.81	0.01	0.28	15.55 (0.00)	(−0.97)–(−0.17)
Emotional Repair > Social Support	0.33	0.12	2.62	0.01	0.40	27.31 (0.00)	(0.08)–(0.57)
Emotional Repair > Lack of Organizational Justice	−0.36	0.14	−2.52	0.01	0.18	8.64 (0.00)	(−0.65)–(−0.08)
**Moderator Effect Psychosomatic Nurses’ Health Problems**							
	Coefficients	SE	t	*p*	R^2^	F (*p*)	Confidence Interval
Emotional Attention > Lack of Organizational Justice	0.16	0.08	2.00	0.05	0.19	5.51 (0.00)	(0.01)–(0.31)

Note: Only moderators considered significant were included.
